# Comparative evaluation of anchorage loss and canine retraction rate using mini-implant anchorage versus conventional molar anchorage

**DOI:** 10.6026/973206300221232

**Published:** 2026-02-28

**Authors:** Sunanda Paul, Md Kalim Ullah, Shahnaz Hamza, Subash Chandra Nayak, Immaneni Pavan Sai Kumar, Sai Sireesha P, Rubeena Naaz, Md Kafeel Ahmed

**Affiliations:** 1 Department of Orthodontics Dentofacial and Orthopaedics, Government Dental College and Hospital, Silchar, Assam, India; 2 Department of Dentistry, Fakhruddin Ali Ahmed Medical College and Hospital, Barpeta, Assam, India; 3 Department of Orthodontics and Dentofacial Orthopaedics, Educare Dental College, Malappuram, Kerala, India; 4 Department of Orthodontics and Dentofacial Orthopaedics, Hi-Tech Dental College and Hospital, Bhubaneswar, Odisha, India; 5 Department of Orthodontics and Dentofacial Orthopaedics, CKS Theja Institute of Dental Sciences and Research, Tirupati, Andhra Pradesh, India; 6 Department of Orthodontics and Dentofacial Orthopaedics, Consultant in Hyderabad, Telangana, India; 7 Dental Surgeon, SRK Dental Clinic, Hyderabad, Telangana, India; 8 Department of Periodontology and Implantology, MNR Dental College and Hospital, Sangareddy, Hyderabad, Telangana, India

**Keywords:** Mini-implant, anchorage loss, canine retraction, temporary anchorage devices (TAD), orthodontic treatment, skeletal anchorage

## Abstract

Orthodontic anchorage control during space closure is critical to prevent unwanted tooth movement, prolong treatment and maintain facial aesthetics. This randomised controlled trial compared mini-implant versus conventional molar anchorage (transpalatal arch) in 40 premolar extraction patients for bilateral canine retraction over 3 months. Mini-implants significantly reduced anchorage loss (0.42±0.31 mm versus 2.18±0.67 mm, p<0.001) compared to conventional anchorage. Canine retraction rates were superior with mini-implants (1.24±0.28 mm/month versus 0.89±0.22 mm/month, p<0.001). Mini-implant anchorage provides superior preservation and faster canine retraction, establishing it as the preferred choice for maximum anchorage needs.

## Background:

During orthodontic therapy, the premolar teeth are often extracted so as to form space in which the crowded dentition is to be aligned or the projecting anterior teeth are to be drawn back. The regulated relocation of teeth into the area of extraction and preservation of the position of anchor teeth are among the most essential issues of the treatment mechanism in such cases [1]. The concept of anchorage, which is defined as the force opposing the undesirable movement of teeth, has been a basic factor when planning orthodontic treatment since the speciality began [2]. The principle of reciprocal anchorage, wherein the movement pattern of the applied force is also experienced on the tooth that is being moved and on the anchor unit, has been identified as a major constraint in the attainment of the intended treatment results. The traditional methods of anchorage have traditionally used the posterior teeth, which are usually molars, as anchor units. The methods have been supported by several instruments such as headgear, transpalatal arches, Nance holding appliances, as well as consolidated posterior segments [3]. In spite of these reinforcing effects, research has always proved that the conventional systems of anchorage are linked to unpredictable levels of anchorage loss, between 1.5 and 4.0 mm in cases of space closure operations [4]. This mesial movement of posterior teeth is not only a decrease in the extent of anterior retraction, but also may result in the occurrence of unwanted alterations in the occlusion and facial profile. The effects of the implementation of temporary anchorage devices (TADs), especially mini-implants or miniscrews, have revolutionised the concept of orthodontic anchorage in the last 20 years [5]. They are relatively tiny (1.2-2.0 mm in diameter, 6-12 mm in length) titanium devices that may be inserted in several different anatomical sites in the alveolar bone and are theoretically independent of the structure of the teeth [6]. The root benefit of mini-implant anchorage is that it does not move in response to orthodontic loads and thus it does not provide or does minimal movement of the opposing teeth. A clinical study of miniboost usage has had a success rate of between 80-95 percent and the variables that affect the success rate are the location of the implant placement, loading and oral hygiene of the patient [7].

The general result of studies comparing skeletal anchorage to conventional methods has been in favour of mini-implants with respect to anchorage preservation, but the size of this benefit differs with different research methodologies and clinical scenarios [8]. Moreover, the effect of anchorage type on the rate of tooth movement has also been considered, with some studies indicating possible faster retraction with skeletal anchorage in contrast to the traditional systems [9]. Although increasing evidence is published in the literature to support the use of mini-implants, several questions still remain on their comparative effectiveness in certain clinical conditions. There has not been a widespread comparison of the rate of canine retraction, which is essential to the efficacy of the treatment, between anchorage systems with standardised methodologies [10]. Moreover, the literature on quantitative measurements of anchorage loss during the particular stage of canine retraction, rather than en masse retraction, is scarce [11]. This difference is clinically significant because canine retraction is a specific stage of treatment that has biomechanical peculiarities. Canine retraction mechanics is based on the biomechanical principles that vary significantly between the mini-implant anchorage system and the conventional anchorage system. In mini-implant anchorage, the retraction force is applied at a skeletal anchor point, which produces a more predictable force system with fewer side effects on adjacent teeth [12]. Conversely, traditional molar anchorage is based on resistance of the posterior segment, which is vulnerable to the concept of differential anchorage, which can lead to diverse levels of movement in anchor units depending on the area of root surfaces and periodontal support [13]. Systematic reviews that have been conducted recently have witnessed the necessity to have well-designed randomised controlled trials between mini-implant anchorage and traditional approaches, especially quantitative variables like anchorage loss and tooth movement rate [14]. Therefore, it is of interest to compare the loss of anchorage and the rate of canine retraction between mini-implant anchorage and traditional molar anchorage in patients who need bilateral first premolar extraction in the maxillary arch.

## Materials and Methods:

## Study design:

The trial was a prospective randomised controlled trial that was done in the Department of Orthodontics.

## Sample size calculation:

The power analysis software was used to determine the sample size based on the data provided in the past studies regarding the mean difference of anchorage loss between mini-implant and conventional anchorage groups. With a difference in anchorage loss of 1.5 mm as the assumed clinically significant difference between the groups, 1.2 mm as the standard deviation, 0.05 as the alpha error and 80 per cent power, 18 participants per group were needed at minimum. A total of 20 subjects per group were recruited to take into consideration the possible drop-outs, allowing the sample size to be 40 patients.

## Participant selection:

The orthodontic outpatient clinic was used to recruit the patients and the following criteria were used:

## Inclusion criteria:

[1] Age between 15 and 30 years

[2] Malocclusion of Class I or Class II Division 1, which necessitates the extraction of both first premolars on the maxillary arch.

[3] Maximum treatment anchorage requirements.

[4] Full set of permanent dentition (no third molar)

[5] Periodontal health and good oral health.

[6] No systemic matters that influence bone metabolism.

[7] No prior orthodontic dental intervention.

## Exclusion criteria:

[1] Craniofacial syndromes, cleft lip/palate.

[2] Extreme skeletal imbalances that necessitate orthognathic surgery.

[3] Maxillofacial history of trauma.

[4] Periodontal or active caries.

[5] Drugs with an action on the bone metabolism (bisphosphonates, corticosteroids)

[6] Poor compliance with patients' expectations.

## Randomisation and group allocation:

Using computer-generated random numbers that are sealed in opaque envelopes, the eligible participants were selected as one of two groups randomly:

[1] Group A (Mini-Implant Anchorage): Mini-implant anchorage to canine retraction was done to 20 patients.

[2] Group B (Conventional Molar Anchorage): Twenty patients were subject to conventional molar anchorage that was reinforced with a transpalatal arch.

## Treatment protocol:

All the patients were attached with 0.022 x 0.028-inch slot pre-adjusted edgewise brackets (MBT prescription) on the maxillary arch. The alignment and levelling were done with the help of the standard archwire sequence: 0.014-inch NiTi, 0.016-inch NiTi, 0.016 x 0.022- inch NiTi and 0.019 x 0.025-inch stainless steel. After the performance of the levelling and alignment, bilateral first premolar extraction was performed under local anaesthesia.

## Group A protocol:

The mini-implants (1.6 mm diameter and 8 mm length, self-drilling titanium alloy) were inserted unilaterally in both the maxillary buccal bone of the alveoli between the roots of the second premolar and first molar (usually about 8-10 mm) relative to the main archwire. Placement was done with local anaesthesia with a manual screwdriver according to standard practice. A straight-away loading was activated with a nickel-titanium closed-coil spring, heightening 150 g of force per side, joined between the mini-implant head and power arms bonded to the brackets on the canines.

## Group B protocol:

A transpalatal arch was made and cemented to the first maxillary molars and then premolar extraction was done. Nickel-titanium closed-coil springs with a force of 150 g per side were used to do the canine retraction, which was hooked to the first molar tubes and operated the arms on the canine brackets. Stainless steel ligature ties were used to consolidate the molars and the second premolars.

## Data collection:

Two time points were used in data collection:

[1] T0 (Baseline): Right before canine retraction is initiated.

[2] T1: Three months following the start of canine retraction.

## The records at different time points were as follows:

[1] Lateral cephalometric radiographs (standardised exposure factors)

[2] Maxillary dental casts (already set in type IV dental stone, poured in as an alginate impression)

[3] Intraoral photographs

## Measurements:

## Cephalometric analysis:

All subsequent cephalometric radiographs later on were digitised and examined on Dolphin Imaging software (version 11.95) with one calibrated examiner. The measured values were the following:

## Anchorage loss:

Horizontal distance movement of the maxillary first molar mesiobuccal cusp tip relative to the pterygoid vertical (PTV) line at T0 and T1.

## Canine retraction:

Horizontal difference in the change of the tip of the maxillary canine cusp towards PTV between T0 and T1.

## Model analysis:

A 3D scanner was used to scan dental casts and the measurements were taken with the help of digital software:

[1] Periodontal distance between the cusp tip of the canine and the mesial surface of the first molar.

[2] Rapidity between the first molar mesial surface and the midpalatal raphe.

## Rate calculation:

The canine retraction rate was determined by dividing the sum of the canine retraction by the observation period (3 months), which was in mm/month.

## Statistical analysis:

The SPSS software was used to analyse the data (version 26.0, IBM Corp., Armonk, NY). The Shapiro-Wilk test was used to test the normality of data distribution. Calculation of descriptive statistics was made to take means, standard deviations and percentages. Continuous variables in the groups were compared using independent samples t-tests. Categorical variables were tested with Chi-square. Intra-examiner reliability had been determined using intraclass correlation coefficients (ICC) on repeated measurements of 10 randomly selected cases after a period of two weeks. A p<0.05 was the preset statistical significance.

## Results:

All 40 enrolled participants completed the study protocol. The mean age of participants was 21.3±4.2 years in Group A and 20.8±3.9 years in Group B, with no significant difference between groups (p=0.692). The gender distribution was similar between groups (Group A: 12 females, 8 males; Group B: 11 females, 9 males; p=0.749). No mini-implant failures occurred during the study period and no significant adverse events were recorded in either group (Table 1). The intra-examiner reliability demonstrated excellent agreement with ICC values ranging from 0.92 to 0.97 for all measurements. The mini-implant group demonstrated significantly less anchorage loss compared to the conventional anchorage group. In Group A, the mean anchorage loss was 0.42±0.31 mm, while in Group B, the mean anchorage loss was 2.18±0.67 mm. This difference was statistically significant (p<0.001). The anchorage loss in the mini-implant group represented a 5.7% loss of the initial extraction space, compared to 30.3% in the conventional group (Table 2). The rate of canine retraction was significantly higher in the mini-implant group compared to the conventional group. Group A demonstrated a mean retraction rate of 1.24±0.28 mm/month, while Group B showed a mean rate of 0.89±0.22 mm/month (p<0.001). Over the three-month observation period, the total canine retraction was 3.72±0.84 mm in Group A and 2.67±0.66 mm in Group B (p<0.001) (Table 2). Detailed cephalometric analysis revealed additional differences between the groups. The maxillary incisor inclination change was greater in Group A (-2.4±1.8 degrees) compared to Group B (-1.1±1.4 degrees), though this difference did not reach statistical significance (p=0.067). The vertical position of the maxillary molars showed minimal changes in both groups, with no significant difference detected (Table 3).

## Discussion:

The findings of this randomised controlled trial have shown that mini-implant anchorage has much better anchorage control than traditional molar anchorage in maxillary canine retraction. The mini-implant group showed a minimum loss of anchorage of 0.42 mm of the anchorage and this is eighty per cent less than the conventional group of 2.18 mm. The findings reject the null hypothesis and prove the clinical benefits of skeletal anchorage to maintain the position of the posterior teeth during space closure surgeries. The loss of anchorage measured on the conventional group is in line with other available literature, which has indicated molar mesial movement of 1.5 to 3.5 mm in the case of canine retraction, although reinforcement appliances were applied [15]. Although the transpalatal arch can offer some resistance to the molar rotation effect and expansion, it can offer minimal resistance to the mesial translation forces applied across the archwire and retraction processes [1]. Conversely, minimum loss of anchorage in the mini-implant group is consistent with research studies, which have established that correctly positioned and loaded mini-implants stand virtually still during orthodontic forces [17]. The large difference in canine-level of retraction between mini-implant anchorages (1.24 mm/month) versus anchorage with 0.89mm (0.89 mm/month) has clinical significance in terms of relevance to the efficiency of treatment. The observation is in line with the research showing skeletal anchorage supports more effective movement of teeth by removing the force dissipation realised when using conventional anchorage systems [18]. When the anchor unit is acted upon and does not move in the desired direction due to the influence of the applied forces the part of the mechanical energy is spent on unwanted tooth movement instead of the desired retraction. The fixation of mini-implants is what makes the entire scope of applied force concentrate on the retraction of the canines. The biomechanical reason that can explain the increased rate of retraction due to mini-implant anchorage can be associated with the ability to provide consistent force delivery when the point of anchorage is stable [19]. In conventional anchorage, the progressive mesial movement of molars changes the force movement and strength with progressive treatment, which may negatively impact the effectiveness of tooth movement. Also, the capability of directly applying force to the canine using the mini- implant without the use of the posterior segment removes the losses to friction experienced in the sliding mechanics of the archwire [20]. Further benefits of mini-implant anchorage observed through the cephalometric analysis were the increase in the incisor retraction during the canine retraction phase. This finding indicates that the rigid anchorage that mini- implants offer can be used to offer more precise retraction of the entire anterior segment and forces are sent through contact point relationships to the incisors [21].

The observed substantial decrease in upper lip protrusion with the use of mini-implants, albeit with a low absolute value, suggests that skeletal anchorage can be used to promote earlier improvements in the soft tissues during the treatment. The 100 per cent success rate of mini-implants in this research is an indication of the care paid to the protocol of placing teeth, the site and the loading parameters. The buccal position in the alveolar bone between the 2nd premolar and the 1st molar roots gives sufficient bone thickness and accessibility [22]. The loading protocol used is immediate loading and the force used was 150 g, which is within the range proven to be safe and effective with mini-implant anchorage [9]. The fact that patient selection did not include patients with poor oral hygiene or other systemic conditions that influence bone metabolism probably helped to attain the promising results. It is interesting to note that the amount of molar tipping in the conventional group (2.8 degrees) was much higher than in the mini-implant group (1.2 degrees). This disparity is due to the ability of the molars to tip mesially due to the action of retraction forces when used as anchor units [23]. These tip movements may disrupt the final occlusion and might need extra treatment time to rectify the same. The slight molar inclination in the mini-implant group maintains the initial molar axial inclination, which makes the finishing phase of treatment much easier. These findings have clinical implications in terms of treatment plan consideration. Mini-implant anchorage has unique benefits in relation to the attainment of treatment goals in severe cases of bimaxillary protrusion or Class II correction, with the extraction of premolars [24]. This is because the preservation of posterior tooth position will guarantee the use of all the available extraction space for anterior retraction to achieve as much profile improvement as possible. In a patient-centred approach, the increase in canine retraction rate as a result of mini-implant anchorage corresponds to a decrease in the total treatment time. Since canine retraction is generally one of the most time-intensive stages of extraction treatment, a 39 per cent faster retraction rate would decrease the duration of this stage considerably [25]. This should, however, be offset against the extra process involved in mini-implant placement and the resultant discomfort on the part of the patient, but the literature has typically indicated that morbidity is not very high with more modern methods of implant placement [26]. The weaknesses of the research are that the observation period was relatively short to cover the canine retraction phase and it does not represent the entire period of treatment and the results. Also, the research was carried out in a controlled clinical setting where standardised procedures were used, which might not be applicable in all practice settings. The limited applicability of the success rates observed may be due to the exclusion of patients with poor oral hygiene or systemic conditions. These findings would be reinforced by future research having more extended periods of follow-ups and having a more varied sample of patients. Economic factors of mini- implant anchorage, such as costs of devices and the costs of placing them, should be balanced with the possible advantages of less time to treat and better results [27]. Analyses of cost-effectiveness that include direct costs as well as time savings value would give useful information in the treatment planning decisions.

## Conclusion:

This randomised controlled trial confirms mini-implant anchorage provides superior anchorage control (80.7% loss reduction: 0.42 versus 2.18 mm) and 39% faster canine retraction (1.24 versus 0.89 mm/month) versus conventional molar anchorage. These findings establish mini-implants as the preferred choice for maximum anchorage cases, enhancing treatment efficiency while preserving anterior tooth position and facial aesthetics. Future longitudinal studies should evaluate long-term outcomes and cost-effectiveness to further optimise clinical decision-making for orthodontic space closure.

## Figures and Tables

**Table 1:** Demographic and baseline characteristics of study groups

**Variable**	**Group A (Mini-Implant) n=20**	**Group B (Conventional) n=20**	**p-value**
Age (years), mean±SD	21.3±4.2	20.8±3.9	0.692
Gender (Female/Male)	8-Dec	9-Nov	0.749
Malocclusion (Class I/Class II)	12-Aug	11-Sep	0.749
Overjet at baseline (mm)	6.8±1.9	7.1±2.1	0.635
Overbite at baseline (mm)	3.2±1.1	3.4±1.3	0.601
ANB angle (degrees)	4.8±1.6	5.1±1.8	0.576
Initial extraction space (mm)	7.4±0.8	7.2±0.9	0.461

**Table 2:** Comparison of anchorage loss and canine retraction between groups

**Variable**	**Group A (Mini-Implant) n=20**	**Group B (Conventional) n=20**	**Mean Difference**	**95% CI**	**p-value**
Anchorage loss (mm)	0.42±0.31	2.18±0.67	1.76	1.43-2.09	<0.001*
Total canine retraction (mm)	3.72±0.84	2.67±0.66	1.05	0.57-1.53	<0.001*
Rate of retraction (mm/month)	1.24±0.28	0.89±0.22	0.35	0.19-0.51	<0.001*
Net space closure (mm)	4.14±0.79	4.85±0.82	0.71	0.19-1.23	0.009*
Anchorage loss (% of space)	5.7±4.2	30.3±9.1	24.6	20.3-28.9	<0.001*
*Statistically significant (p<0.05)

**Table 3:** Cephalometric changes during canine retraction phase

**Variable**	**Group A (Mini-Implant)**	**Group B (Conventional)**	**p-value**
**Molar Position Changes**			
Horizontal (to PTV) (mm)	0.42±0.31	2.18±0.67	<0.001*
Vertical (to palatal plane) (mm)	0.18±0.24	0.31±0.28	0.124
Molar tip (degrees)	1.2±0.9	2.8±1.4	<0.001*
**Canine Position Changes**			
Horizontal (to PTV) (mm)	-3.72±0.84	-2.67±0.66	<0.001*
Vertical (to palatal plane) (mm)	0.22±0.31	0.28±0.34	0.561
Canine tip (degrees)	-4.2±2.1	-3.1±1.8	0.083
**Incisor Changes**			
U1 to SN (degrees)	-2.4±1.8	-1.1±1.4	0.015*
U1 to NA (mm)	-0.8±0.6	-0.4±0.5	0.027*
**Soft Tissue Changes**			
Upper lip to E-line (mm)	-0.4±0.3	-0.2±0.2	0.018*
*Statistically significant (p<0.05);
Negative values indicate retraction/reduction
